# Time to add two new disorders to neuropsychiatric aspects of Parkinson’s disease

**DOI:** 10.1016/j.prdoa.2025.100358

**Published:** 2025-06-15

**Authors:** Stefano Luca Sensi, Matteo Alessandro De Rosa, Mirella Russo, Astrid Thomas, Marco Onofrj

**Affiliations:** a Department of Neuroscience, Imaging and Clinical Sciences, “G. d'Annunzio” University of Chieti-Pescara, 66100 Chieti, Italy; b Center for Advanced Studies and Technology (CAST), “G. d’Annunzio” University of Chieti-Pescara, Chieti, Italy; c Neurology Institute, SS. Annunziata University Hospital, “G. d’Annunzio” University of Chieti-Pescara, Chieti, Italy; d Institute for Advanced Biomedical Technologies (ITAB), “G. d’Annunzio” University of Chieti-Pescara, Chieti, Italy

**Keywords:** Parkinson’s Disease, Neuropsychiatric Disorders, Somatic Symptom Disorders, Bipolar Spectrum Disorders

## Abstract

Recent reviews have explored Parkinson’s Disease (PD) through psychiatric lenses. However, emerging literature allows to go beyond psychosis, depression, and impulse control disorders. Two key emerging themes are prominent: firstly, studies accrued in the past 13 years challenge prevailing views by demonstrating Somatic Symptom Disorders/Somatoform Disorders (SSD-SD) significantly participate in the PD phenotype, with relevant treatment implications. Secondly, epidemiological data reveal a link between Bipolar Spectrum Disorders (BSD) and PD, prompting a reconsideration of Impulse Control Disorder/Hedonistic Homeostatic Dysregulation (ICD/HHD) in PD. Furthermore, behaviors akin to BSD symptoms during manic states are observed in PD, suggesting shared neurobiological underpinnings.

Our proposed review tackles these themes, dissecting the impact of SSD-SD on PD and highlighting the BSD-PD convergence. This approach has implications for tailored psychotherapeutic techniques targeting psychosomatic and functional neurologic disorders within the context of PD care. Moreover, recognizing the blurred lines between neurology and psychiatry in PD underscores the importance of a holistic, interdisciplinary approach.

As these findings challenge established assumptions and offer new avenues for patient care, we must promote our understanding of PD’s intricate neuropsychiatric dimensions.

Recent reviews such as the landmark ones by Ffytche et al. [1], Weintraub et al. [2], and Pagonabarraga et al. [3] have discussed the psychiatric aspects of Parkinson’s disease (PD), but it is becoming clear that the neuropsychiatric disorders reported in recent literature encompass more than just psychosis with hallucinations and delusions, depression, and impulse control disorders (ICD) mentioned in these reviews.

Two major issues have emerged:

(1) Over the past 13 years, several papers [[4], [5], [6], [7], [8], [9], [10], [11], [12], [13], [14], [15], [16], [17], [18]] have concurred in demonstrating that the category of Somatic Symptom disorders/Somatoform disorders (SSD-SD, as classified in the DSM-5) represent an important phenotype of PD, significantly impacting therapeutic strategies. Before the systematic studies [[4], [5], [6], [7], [8], [9], [10], [11], [12], [13], [14], [15], [16], [17], [18]], alexithymia, a trait linked to SSD-SD, was noted to be common in PD [[19], [20], [21], [22]].
(2) In the past three years, scientific and epidemiological studies involving thousands of patients have revealed that Bipolar Spectrum Disorders (BSD) are a strong predictor of PD occurrence, multiplying the odds ratio by 3.5 to 6.7 [[23], [24], [25], [26], [27], [28], [29], [30], [31], [32], [33], [34]]. This impacts treatment approaches and outcome expectations, as well as calls for a reassessment of current assumptions regarding drug treatments.

In the proposed review paper, we intend to analyze and dwell on these two issues as follows:

## 1 Somatoform disorders in Parkinson’s disease

SSD-SD includes manifestations historically defined as hypochondria, Briquet's syndrome, hysteria, conversion disorders, somatoform disorders, psychosomatic disorders, and psychogenic disorders. It should be noted that the term Somatoform Disorders was used in the DSM-IV-TR classification but then changed to Somatic Symptom Disorders (SSD) in the DSM-5 and DSM-5-TR (see Table 1). Several psychiatric studies and the International Classification of Diseases-11 (ICD-11) still employ this term, which was also used in our previous studies and by several studies corroborating our findings [[4], [5], [6], [7]].

**Table 1:** Classification and examples of psychiatric symptoms in Parkinson’s Disease.

Somatic Symptom and Related Disorders (adapted from DSM-5)	
▪ Somatic Symptom Disorder	
▪ Illness Anxiety Disorder	
▪ Conversion Disorder (Functional Neurological Symptom Disorder) e.g., functional tremors, palsy, dysphagia, dystonia, psychogenic nonepileptic seizures, gait disorders	
▪ Factitious Disorder	
Disruptive, Impulse-Control, and Conduct Disorders (adapted from DSM-5)	
▪ Other Specified Disruptive, Impulse-Control, and Conduct Disorder (DSM-5) e.g.:	
o Gambling	
o Hypersexuality (including paraphilia)	
o Compulsive shopping	
o Binge-eating disorder, bulimia	
o Punding	
o Obsessive hobby pursuing	
Bipolar and Related Disorders	
DSM-5	**Akiskal’s classification**
▪ Bipolar I Disorder	▪ Bipolar I: Full-Blown Mania
	▪ Bipolar I ½: Depression With Protracted Hypomania
▪ Bipolar II Disorder	▪ Bipolar II: Depression With Hypomania
▪ Cyclothymic Disorder	▪ Bipolar II ½: Cyclothymic Depressions
▪ Substance/Medication-Induced Bipolar and Related Disorder	▪ Bipolar III: Antidepressant-Associated Hypomania
	▪ Bipolar III ½: Bipolarity Masked-And Unmasked-By Stimulant Abuse
	▪ Bipolar IV: Hyperthymic Depression
	▪ Bipolar V: Recurrent Depression that is Admixed with Dysphoric Hypomania
▪ Bipolar and Related Disorder Due to Another Medical Condition	▪ Bipolar VI: Late-Onset Depression With Mixed Mood Features, Progressing To Dementia
▪ Other Specified Bipolar and Related Disorder	

Abbreviations: DSM-5 = Diagnostic and Statistical Manual of Mental Disorders – 5th edition.

It should be stressed that in patients with Parkinson’s disease, these disorders precede the onset of motor symptoms by years and, in susceptible patients, are primarily associated with the early stages of the disease [[7], [8], [9], [10]]. They become less evident, but not always, when cognitive decline is marked. When motor symptoms have already appeared, the disorders manifest as gastrointestinal, genitourinary, and joint discomforts, along with locally undefined pain characteristics of SSD [11,12]. They are associated with obsessive concerns for the severity of these symptoms and, in parallel, a lack of awareness (scotomization) of the motor symptoms specific to PD. Expressed concerns about abdominal disorders, bloating, dyspepsia, and various pains are not affected and do not follow the pharmacokinetics of L-Dopa (unlike pain associated with PD). They are the expression of intolerance to medications that cannot be explained based on pathophysiology or pharmacology. Patients, for instance, may exhibit a delusional intolerance to a specific commercial preparation of a given compound. They may lament complications not justified by underlying pathophysiological processes, such as dyskinesias occurring during OFF periods when therapy for motor symptoms has already been introduced [8].

Patients also exhibit somatoform manifestations or conversion disorders (or Functional Neurologic Disorders −FND (Table 1), the term is now accepted, in substitution of conversion, in the last revision of DSM-5-TR) years before the appearance of PD motor symptoms [6,8,9,13,14,18]. In some cases, motor manifestations of PD, such as distractible and unexplained tremors or paralysis, are described. Moreover, there is also a long history of disorders classified as fibromyalgia, chronic fatigue syndrome, or painful syndromes without pathological substrates, such as reflex sympathetic dystrophy, episodes of globus pharyngis, or pseudo crises/psychogenic seizures [7,12,16].

According to some authors, the prevalence of these disorders is around 7–20 % of PD patients [7,15], while others indicate 40–59 % [4,6,16]. Psychopathological evaluations of these patients often reveal an a-critical insight regarding these disorders, sometimes with psychotic elements [7,16,17]; also, alexithymia is a trait reported to predispose to SSD-SD and is common in PD [[19], [20], [21], [22]]. Thus, it has been hypothesized that, in some PD patients, SSD can be interpreted as signs of true somatic hallucinations [14]. In some PD cases where cognitive decline has already ensued, somatic delusions emerge. These are characterized by hallucinatory sensations of deformity, delusions of parasitic infestations or multiple allergies, and delusions of having necrotic parts of one's body, respectively, Ekbom and Cotard syndromes in classifications of rare psychiatric syndromes [2,7,12].

The overlap of psychosomatic disorders sometimes makes it challenging to accept chronic therapy, leading to the development of unconscious therapy refusal and the production of disorders attributed to therapy that cannot be explained by the drug's molecular mechanism or are entirely incongruous with the drug's duration.

## 2 Bipolar disorders associated with Parkinson’s disease

The recent epidemiological evidence that Bipolar Disorders are linked to PD [[23], [24], [25], [26], [27], [28], [29], [30], [31], [32], [33], [34], [35]] challenges dominant assumptions on the gist of impulse control disorder/hedonistic homeostatic dysregulation (ICD/HHD) in PD patients. ICD/HHDs include compulsive gambling, hypersexuality (including paraphilic manifestations), compulsive shopping, bulimia, as well as other compulsive or coercive behaviors such as aggressive driving, obsession with bricolage, exercise, house cleaning, or gardening [[36], [37], [38], [39]] (see also Table 1). The latter disturbance is also called punding (a Danish term indicating behavior induced by amphetamine use). In PD patients, these manifestations have been observed in coincidence with the introduction of second-generation dopamine agonists (DAs), such as ropinirole, pramipexole, and rotigotine, but had also been previously described with the use of first-generation, ergolinic, DAs (i.e., bromocriptine, cabergoline, pergolide) or with the introduction of L-Dopa. According to some studies, DAs precipitate ICDs, whose incidence increases with DA exposure from 5-7 % to 18 % [36], or up to 40–50 % [[37], [38], [39]]. These disorders have accrued wide media coverage, leading to an outbreak of litigations and class actions that have virtually ended research on DAs. However, in carriers of *PARK1*, *PRKN*, *LRRK2*, or *GBA* mutations, ICD/HHD are very common and appear before the onset of motor symptoms and, of course, before exposure to DA or L-Dopa therapies [[40], [41], [42], [43]], an indirect proof of the underlying neural and biological substrates of these disorders. In PD patients, several risk factors have been acknowledged for ICDs, including male sex (particularly for hyper sexuality), younger onset age (which is linked to genetic etiology), smoking habit, mood disorders like anxiety and depression, as well as personality traits of novelty seeking, impulsivity, propension to obsessive–compulsive behaviors [44]. To assess any differences in ICD prevalences and features between *parkin* and *non-parkin* PD, Morgante et al. [45] carried out a case-control study. Although the two groups did not differ in terms of ICD frequency, the *parkin* group showed greater severity of the symptoms and a higher propensity for binge eating, punding, and compulsive shopping. A prominent and selective degeneration of the caudate nucleus, which is part of the fronto-striatal-limbic circuitry along with the orbitofrontal cortices, has been observed in *parkin*-related PD patients, compared to other forms, and may precociously interfere with the reward circuitry, thus leading to ICD [46].

It must be emphasized that identical altered behaviors are observed in patients with bipolar disorders during manic/hypomanic or mixed states. According to the DSM or International Classification of Diseases-11, these behaviors constitute a core diagnostic element for BSD.

Despite the outstanding similarity between ICD/HHDs of PD and BSD manifestations, the similarity has been largely overlooked, possibly because the clinicians reporting them (i.e., neurologists) were not psychiatrists. Indeed, the historical attitude of neurologists has been that all cognitive or psychiatric disorders in PD were a casual epidemiological coincidence, although rarely formally stated [47,48].

However, recent literature has demonstrated, through studies on large populations, that PD occurs much more frequently (OR multiplied by 4 or more) in patients who presented with bipolar disorders [27,28]. This indicates that common dysfunctions of neural circuits occur in both pathologies and suggests new hypotheses to be pursued. We will discuss the overlapping neurochemical and neural substrates of BSD and synucleinopathies in detail.

There is also an important practical aspect. The presence of bipolar spectrum disorders is a predictor of poor outcome of advanced therapies such as deep brain stimulation [29,[49], [50], [51]], as authors of early guidelines already showed 17 years before recent epidemiological studies [[49], [50], [51]]. The PD-bipolar disorder phenotype, moreover, predicts the appearance of ICD/HHDs, and Dopamine Withdrawal Syndrome (DAWS), predicts psychosis and SSD. Therefore, it must be considered by careful specialists set to manage this kind of patient.

The evidence also raises the issue of how much societal damage has been caused by the media campaigns that drove class actions on dopamine agonist treatments, unproperly dealing with medical conditions that required adequate epidemiological and scientific clarifications.

Since the presence of bipolar disorders is a powerful predictive factor for the appearance of Parkinson’s disease, the lack of rationale of many media oversimplifications produced in the past and many medicolegal conclusions must be reconsidered.

## 3 Therapeutic implications

The recent literature shows therefore that SSD-SD are common in PD [[4], [5], [6], [7], [8], [9], [10], [11], [12], [13], [14], [15], [16], [17], [18]] and that bipolar disorders are linked to PD [[23], [24], [25], [26], [27], [28], [29], [30], [31], [32], [33], [34], [35]], earlier epidemiological studies and anecdotal reports had already underlined these associations [[52], [53], [54], [55], [56], [57], [58], [59], [60], [61]].

The treatment of SSD and FND mostly relies on psychotherapy and physical therapy [62]. Randomized placebo-controlled trials have not shown that any specific antidepressant is uniquely effective for treating this disorder, however antidepressant and anxiolytic therapies could improve the outcomes if a comorbid depression or anxiety is observed [63]. Evidence of SSD and BSD in PD suggests that the range of psychotherapies offered to PD patients should be expanded to include techniques applied to these two psychiatric disorders [64,65].

We suggest that short psychotherapy techniques devised for psychosomatic and functional neurologic disorders should be applied in PD treatment settings. These include short-term psychodynamic psychotherapy, cognitive behavioral therapy, treatment of anxiety and physical symptoms, and group psychoeducation. Patient support organizations’ anecdotal or occasional observations already provide preliminary evidence of possible benefits.

The implications of a PD diagnosis in bipolar patients – *and vice versa* − are wider. The main mood stabilizer, i.e. lithium, has a negative impact mainly on motor performance and could worsen tremors [66]. Valproate can also worsen parkinsonian symptoms [67], while a lesser impact on motor aspects is typically observed for carbamazepine, which has however been also linked to acute dystonia and chorea onset in the general population [68,69]. Although infrequently, motor complications have been described with the use of lamotrigine e.g., dyskinesia, chorea, action and rest tremors [69]. Antidepressants, particularly SSRIs and vortioxetine, can be safely used to address depressive phases and symptoms in PD patients [70]. To treat acute manic phases, neuroleptics and antipsychotics with a higher anti-dopaminergic impact should be avoided if feasible, while second-generation antipsychotics like clozapine and quetiapine show better tolerability [71].

Therefore, an alliance between psychotherapeutic and physical therapy techniques seems better suited to address the needs of patients who share the more complex aspects of PD rather than classic physiotherapy. Moreover, the recent findings highlight the need to bridge the gap between neurology and psychiatry in the interest of patients with neurodegenerative disorders. The neuropsychiatric aspects of these disorders also have a heuristic relevance for understanding different mental disorders.

## CRediT authorship contribution statement

**Stefano Luca Sensi:** Writing – original draft, Conceptualization. **Matteo Alessandro De Rosa:** Writing – review & editing. **Mirella Russo:** Writing – review & editing. **Astrid Thomas:** Writing – review & editing. **Marco Onofrj:** Writing – original draft, Conceptualization.

## Declaration of competing interest

The authors declare that they have no known competing financial interests or personal relationships that could have appeared to influence the work reported in this paper.
